# Gabapentin and Diclofenac Reduce Opioid Consumption in Patients Undergoing Tonsillectomy: A Result of Altered CNS Drug Delivery?

**DOI:** 10.5812/atr.11011

**Published:** 2013-08-01

**Authors:** Patrick T. Ronaldson, Thomas P. Davis

**Affiliations:** 1 Department of Medical Pharmacology, University of Arizona College of Medicine, Tucson, USA

**Keywords:** Blood-Brain Barrier, CNS Drug Delivery, Opioid Analgesic, Transporters

Dear Editor,

We have read with great interest the article by Mogadam and colleagues on utilization of gabapentin and diclofenac for management of post-operative pain in patients undergoing tonsillectomy (1). Perhaps the most intriguing aspect of this study was the observation that pre-operative administration of gabapentin or diclofenac resulted in reduced post-operative utilization of meperidine, an opioid analgesic. Optimal therapeutic efficacy of opioids requires that they cross the blood-brain barrier (BBB) and attain effective concentrations in the CNS (2). CNS delivery of opioids is determined by putative membrane transporters localized to the BBB endothelium (3, 4). Both pathophysiological factors (i.e., inflammatory signaling in pain) and pharmacological factors (i.e., use of ancillary pain medications) can modulate mechanisms involved in BBB opioid transport, an effect that can cause profound changes in CNS delivery of traditional opioids (i.e., morphine, meperidine). In fact, our research group has demonstrated that painful and/or inflammatory stimuli in the periphery can significantly change transport mechanisms for opioids at the BBB such as the drug efflux transporter P-glycoprotein (P-gp) (5) and the drug influx transporter organic anion transporting polypeptide 1a4 (6). Of particular note, we have also shown that diclofenac itself can attenuate pain-induced changes in BBB transporter activity (6). Our data suggest that a modulation in post-operative meperidine efficacy may, in part, be the result of altered CNS opioid delivery induced by diclofenac. An additional factor to consider is that some ancillary pain medications may act as chemical inhibitors of the same BBB transporters. In the context of the study by Mogadam and colleagues, this effect may involve the critical BBB efflux transporter P-gp. Specifically, gabapentin is a known P-gp inhibitor (7) while in vitro studies have suggested that meperidine is a P-gp transport substrate (8). Although in vivo studies in mdr1a knockout mice showed no difference in meperidine antinociception (9), a direct analysis of P-gp-mediated transport of meperidine in intact laboratory animals has not been undertaken. Furthermore, this study measured analgesic efficacy using only tail-pinch, a technique that may not have been sensitive enough to detect differences in meperidine analgesia between P-gp-deficient and P-gp-competent mice. Nonetheless, it is highly plausible that gabapentin blocked P-gp-mediated efflux transport at the BBB, an effect that effectively increased CNS meperidine delivery with the clinical manifestation of reduced opioid consumption in the post-operative period. Overall, results obtained from basic science studies of our laboratory and others point towards an explanation of modified post-operative opioid efficacy in terms of altered BBB transport and/or CNS delivery of meperidine induced by ancillary pain medications. Additionally, the paper by Mogadam and colleagues emphasizes the absolute importance of novel translational studies aimed at understanding specific mechanisms involved in CNS opioid delivery, opioid efficacy, and/or drug-opioid interactions.
